# Carcinoid Syndrome-Induced Ventricular Tachycardia

**DOI:** 10.1155/2016/9142598

**Published:** 2016-03-21

**Authors:** Austin B. Rupp, Abdulmohsin Ahmadjee, Jack H. Morshedzadeh, Ravi Ranjan

**Affiliations:** ^1^Department of Medicine, School of Medicine, University of Utah, Salt Lake City, UT 84132, USA; ^2^Department of Medicine, School of Medicine, King Abdulaziz University, P.O. Box 80215, Jeddah 21589, Saudi Arabia; ^3^Division of Cardiovascular Medicine, School of Medicine, University of Utah, Salt Lake City, UT 84132, USA

## Abstract

*Introduction.* Carcinoid tumors are rare neuroendocrine malignancies that secrete multiple bioactive substances. These bioactive substances are responsible for the carcinoid syndrome characterized by diarrhea, flushing, syncope, and right-sided valvular heart disease. Previous case reports have described carcinoid syndrome associated with coronary vasospasm and the well-characterized carcinoid heart disease.* Case.* Our patient is a 73-year-old female with complex past medical history most notable for metastatic carcinoid tumors diagnosed in 2013-05. She initially presented in 2014-09 with syncope and dizziness associated with sinus pause on an event monitor. She received a pacemaker given normal left ventricular function and was discharged. However, she was readmitted with similar symptoms corresponding to multiple episodes of ventricular tachycardia. She was started on high-dose beta blockade and has had no recurrence of arrhythmia over a follow-up period of 12 months.* Conclusion.* We hypothesize that the patient's ventricular tachycardia was mediated by the multiple bioactive substances secreted by her carcinoid tumors. Her carcinoid tumor biomarkers were elevated and other explanations for arrhythmia were investigated and ruled out. To our knowledge, this is the first case of ventricular tachycardia mediated by carcinoid syndrome and suppressed by beta-blocker. Further investigation into this relationship is needed.

## 1. Introduction

Carcinoid tumors are rare neuroendocrine malignancies that arise from neural crest cells and release a variety of bioactive substances, of which serotonin, histamine, tachykinins, kallikrein, and prostaglandins are thought to be the most prominent. These vasoactive hormones are responsible for the well-described carcinoid syndrome, which is characterized by cutaneous flushing, bronchospasm, diarrhea, and right-sided cardiac disease. Once carcinoid tumors metastasize to the liver or outside the gastrointestinal tract, these vasoactive substances are no longer metabolized by the liver and can cause the classic symptoms of carcinoid syndrome [1]. The usual cardiac manifestations of carcinoid syndrome are well described and include tricuspid and pulmonary valve disease leading to right-sided cardiac dilatation and ultimately right-sided heart failure. The development of carcinoid heart disease occurs in about 50% of patients with carcinoid syndrome and is associated with a poor prognosis [2, 3]. Several case reports have also shown serotonin-related coronary artery vasospasm associated with carcinoid syndrome [4–6]. There is also one reported case of carcinoid syndrome causing ventricular tachycardia in a canine subject [7]. Here, we report a case of ventricular tachycardia presumptively mediated by carcinoid syndrome.

## 2. Case

Our patient is a 73-year-old white female with complicated past medical history including breast cancer for which she underwent lumpectomy and chemotherapy in 2008, hypertension, hyperlipidemia, hypothyroidism, osteoarthritis, and ophthalmologic disease s/p left prosthetic eye placement. She was also diagnosed with metastatic carcinoid tumor after presenting with persistent palpitations, syncope, light-headedness, nausea, vomiting, and abdominal pain in 2013-01. Following exploratory laparotomy with small bowel resection and biopsy of the liver (positive for metastatic carcinoid tumor) in 2013-05, she was noted to have nonsustained atrial fibrillation, for which she established care with a cardiologist. She underwent left and right heart catheterization in 2013-07 that showed no coronary artery disease and minimally elevated pulmonary artery pressure (mean 31 mm Hg) and PCWP (16 mm Hg). An echocardiogram was also performed which showed hyperdynamic left ventricular (LV) function with EF 71%, mild-moderate mitral regurgitation (MR), and apical LV hypokinesis. MRI in 2013-08 showed normal EF and wall motion and absence of fibrosis or scar. Echo was repeated in 2014-02, which again showed normal left ventricular function. She continued to complain of syncopal events and ambulatory monitoring did not show any coinciding arrhythmia, although there were sinus pauses, generally occurring after octreotide injections. Octreotide was switched to lanreotide, and she did experience improvement in her syncopal episodes. Given her recurrent syncope and sinus pauses, the patient had a dual chamber pacemaker placed on 2014-09-09 that was complicated by a carcinoid crisis despite being on an octreotide drip. Her course was relatively uncomplicated following this event, and she was discharged on 2014-09-11.

**Figure 1:**
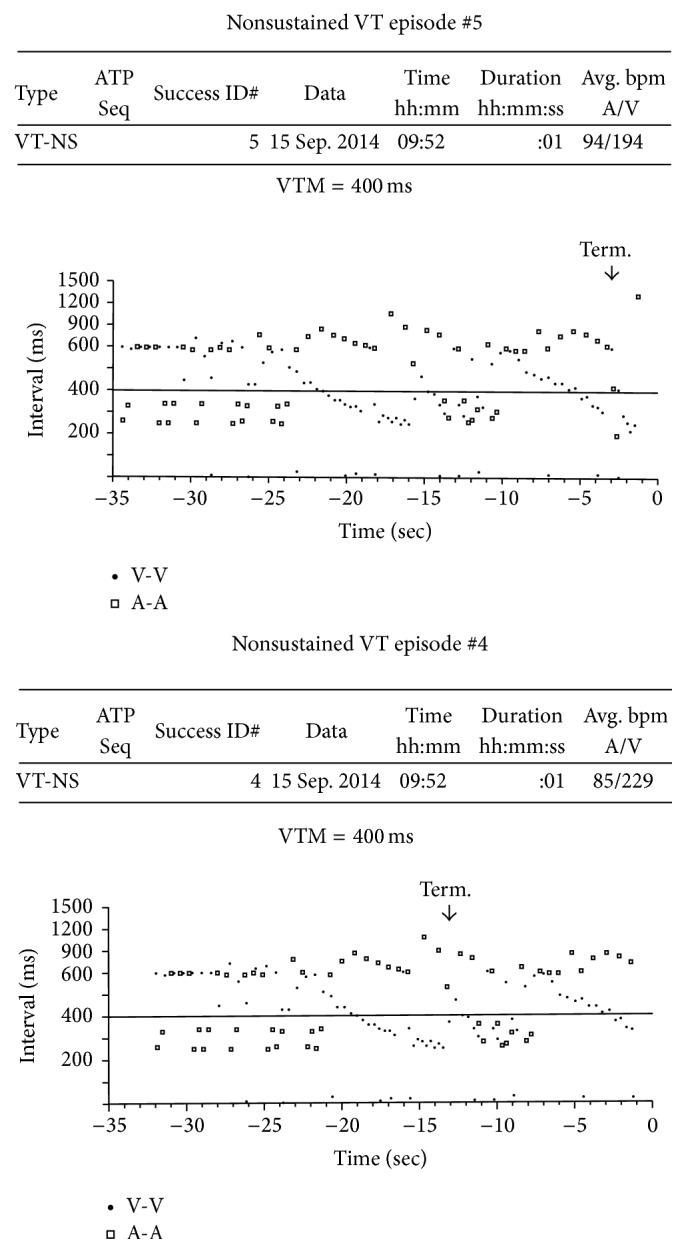
Cycle length plots from patient's device showing ventricular tachycardia (more ventricular vents compared to atrial events) corresponding to patient's syncope and dizziness.

Unfortunately, our patient was readmitted on 2014-09-15 after being found unresponsive at home. She was taken to an outside hospital where she was intubated for acute hypoxic respiratory failure and transferred to our facility. After extubation and upon further questioning, she endorsed two episodes of palpitations and syncope on day prior to admission for which she was given octreotide injections that relieved her symptoms. On the morning of admission, she was lying down and reading a book when she felt similar symptoms with light-headedness, palpitations, and shortness of breath. She called her daughter who found her unresponsive and cyanotic. Emergency personnel were called and she was taken to the hospital. On arrival at our institution she was sedated, and her physical exam was significant for the presence of an endotracheal tube. Cardiopulmonary examination was normal. Laboratory work-up was significant for chronic anemia and thrombocytopenia, troponin of 0.17, and hypoalbuminemia. Creatinine, magnesium, thyroid stimulating hormone, and corrected calcium were all within normal limits (or had been checked within the month preceding admission and were within normal limits). Chest radiograph showed moderate pulmonary edema and head CT was negative for acute intracranial processes. Electrocardiogram was significant for a chronic left bundle branch block (QRS 128 msec), normal sinus rhythm, normal axis, and QTc of 500 milliseconds. The patient was initially treated with antibiotics for presumed aspiration pneumonia and further work-up was pursued. Shortly after admission, the patient was extubated and had no complaints other than dysphonia and sore throat. She denied chest pain following extubation and in association with her presentation. Further work-up was significant for downtrending troponins and negative infectious work-up. Echocardiogram on 2015-09-16 was significant for LVEF 70%. She was also found to have elevated levels of plasma metanephrines and urinary 5-HIAA, increased from previous studies. Most notably, pacemaker interrogation revealed four episodes of ventricular tachycardia. The ventricular tachycardia episodes evolved over 8-9 seconds with a ventricular rate of approximately 200 beats per minute and then spontaneously terminated (Figure 1). The episodes correlated with the patient's syncopal symptoms. As aforementioned, she was extubated, antibiotics were stopped after negative infectious work-up, and consultation with endocrinology and oncology was obtained. We feel that her ventricular tachycardia was due to carcinoid crisis with excessive secretion of multiple bioactive substances, as the episodes were brief and had decreasing cycle lengths pointing to an automatic focus. After discussion with her and her family, ICD implantation was not pursued and she was started on high-dose beta blockade with metoprolol (25 mg every six hours and uptitrated to 200 mg daily by mouth). The high-dose metoprolol adequately suppressed her ventricular tachycardia and she has not had any further recurrences over a follow-up period of approximately 12 months.

## 3. Discussion

Here, we present the case of a 73-year-old female with a complex past medical history most notable for metastatic carcinoid tumors and carcinoid syndrome. She presented with syncope and was found to have multiple episodes of ventricular tachycardia that correlated with her syncopal episodes. Work-up did not reveal any cause for the ventricular tachycardia, and we hypothesize that carcinoid syndrome caused her arrhythmia. She did not have any evidence of ischemia, significant electrolyte abnormality, or advancing structural cardiac disease. She did have conduction abnormalities (rare atrial fibrillation, LBBB, and sinus pauses) previously, but these were not significantly changed from previous and would not account for her syncopal symptoms. Additionally, her QTc was 500 msec when she was admitted with episodes of syncope. However, this was essentially unchanged from prior measurements and when corrected for the presence of LBBB was calculated at 438 msec [8]. While QT prolongation and octreotide potentially predisposed her to arrhythmia, we believe her carcinoid syndrome was the inciting event. Supporting this hypothesis is the fact that markers of her carcinoid syndrome (urinary 5-HIAA and plasma metanephrines) were increased from previous levels.

Presumptively, episodes of carcinoid crises caused increased production of typical vasoactive substances, leading to sympathetic overactivation, increased cardiac myocyte excitability, and ventricular tachycardia. Treatment with metoprolol negated this sympathetic activation and suppressed the arrhythmia. She has not had any recurrences of ventricular tachycardia since initiation of metoprolol. She does continue to experience symptoms of carcinoid crises with wheezing, flushing, dizziness, and dyspnea but has not had VT on pacemaker interrogations.

To our knowledge, this is the first reported instance of carcinoid syndrome associated with ventricular tachycardia without evidence of myocardial ischemia or typical carcinoid heart disease. We believe this potential association is important to acknowledge, as ventricular tachycardia is a life-threatening arrhythmia that requires prompt intervention. Further exploration of associations between carcinoid tumors, their vasoactive products, sympathetic activation, and arrhythmia, particularly ventricular tachycardia, is necessary to better define these relationships and mechanisms.
